# Clinic of Professor Dunglison

**Published:** 1845-03

**Authors:** Samuel G. White

**Affiliations:** Georgia


### CLINICAL LECTURES AND REPORTS.

### PHILADELPHIA HOSPITAL.

*Saturday, December* 21, 1844.

### CLINIC OF PROFESSOR DUNGLISON.

Reported by Mr. Samuel G. White, of Georgia.

The two cases of INTERMITTENT FEVER, reported at the last lecture, Professor Dunglison stated, had recovered, and been discharged. They were cured of the intermittent, but some enlargement of the spleen remains in one of the cases, which was yielding, and will pass away in course of time.

The subject of nutrition having engaged the medical class at Jefferson Medical College during the week, the lecturer took up the consideration of

SCROFULOSIS, tuberculosis having been investigated already.

This is a subject rarely selected for clinical remarks, and yet it is one on the true nature of which vague notions are entertained.

Between scrofulosis and tuberculosis there is much analogy, but they can scarcely, perhaps, be considered altogether identical. Some authors, however, regard them so; the principal apparent difference being in the tissues involved. This view derives confirmation from microscopical investigations; for it is found that the matter of tubercle does not differ from that of a scrofulous abscess. The ages in which they occur are, however, different; for whilst scrofulosis is chiefly confined to children, tuberculosis is mainly a disease of adolescence and virility; and although scrofula is sometimes developed in old age, it will usually be found, on examination, that its foundation was laid in early life. Moreover, an individual may exhibit every sign of a scrofulous habit, and yet be free from any distinct evidence of tuberculosis. It must be admitted, therefore, that the two diseases do differ in some respects, although the remarks made in this lecture may be considered applicable to both affections.

Scrofulosis has been variously defined by different writers. Some consider its main feature to be an indolent enlargement of the glands of the neck, with development of the upper lip, fair skin, light hair and eyes, &c. But it need scarcely be remarked, that this is an exceedingly limited view of the disease, and even objectionable, inasmuch as the appearances indicated may be absent in marked cases of scrofulous habit, and present in other diseases in no way connected with scrofula.

It is admitted by all, that tumefaction and suppuration of the cervical ganglia are symptoms of scrofulosis, but it is something more than this. The inflamed gland is simply an expression of the disease, and not the disease itself: and it is not the only expression, for caries, morbus coxarius, &c., are also evidences of this diathesis.

But inflamed or suppurating glands are not confined to scrofulous persons; they may and do occur in healthy individuals, whenever nutritive irritation exists so as to involve the lymphatic ganglia, between its seat and the thoracic duct. Of this, we often see examples in children affected with irritation of the scalp, giving rise to enlargement of the glands of the neck,—as well as in cases of inflamed inguinal, axillary or mesenteric ganglia, when there exists any source of irritation in the upper or lower extremities, or in the mucous lining of the small intestines. These enlarged glands, in a healthy individual, may also go on to suppuration, but the pus formed is—to use a surgical term of doubtful propriety—'laudable,' and it terminates in health; but if there exists a scrofulous habit, different results will ensue; the gland increases in size, its surface becomes red, then bluish, and soon fluctuation is detected; and if not interfered with, ulceration of the integuments succeeds, accompanied by a discharge of cheese-like pus, followed by an unsightly cicatrix.

These definitions of scrofula, as before remarked, are very imperfect. They enumerate the expressions, but do not define the disease. Scrofulosis, the professor observed, is not a local affection. It is a disease of the blood, and of the entire system of nutrition. Analysis of the blood, drawn from a scrofulous individual, shows a diminution in the ratio of red corpuscles; and although there is an increase in the proportion of the fibrinous constituent, it is of an inferior degree of organizability, differing in this particular from the blood of inflammation. Owing to this imperfect organizability of the fibrinous element, the exudation or pus corpuscles, instead of being of the healthy character, are 'cacoplastic,' or even completely 'aplastic.'

The blood of scrofulosis contains a larger quantity of albumen and less fibrin than that of inflammation, and albumen is less organizable than fibrin, which is, in fact, the only self-organizable material. It should be recollected, that in the healthy execution of the functions albumen is being constantly converted into fibrin; and it can be readily conceived, that if this process be imperfectly performed, the fibrin will be of an inferior quality, and the ratio of the albuminous constituent of the blood may be augmented. In the diseased condition, then, under consideration, as also in tuberculosis, the organic functions appear to be diminished in energy, and the fibrinous element of the vital fluid is endowed with imperfect organizable power, whilst the albuminous element is increased in quantity.

If, then, the blood and the nutritive function be diseased, how imperfect must be the definition, which considers scrofulosis a simple glandular malady! Is it not rather a cachectic condition of the whole system;—a disease of the system of nutrition?

The patient, before the class, did not exhibit much enlargement of the cervical ganglia, but presented every evidence of a highly strumous diathesis.

In both tuberculosis and scrofulosis there is a diathesis, communicating a predisposition, which is developed by exciting causes, that, in a healthy individual, would have no such effect. We may, then, have a scrofulous as we have a tuberculous diathesis.

The case under consideration presents a disposition to the development of irregular inflammatory action in various parts of the system, which must be referred to some peculiarity of constitution. Sometime since, she was much harassed by hyperæsthesis or a preternatural sensitiveness of the whole surface, conjoined with inflammation and extreme tenderness of the scalp, for which the tincture of iodine was employed as a topical application, with much benefit.

The lecturer regards this disease and tuberculosis to be, so far as any diseases are, hereditary; a predisposition being transmitted from parent to child. But, although there is generally this hereditary tendency, it cannot be denied, that it may be developed, under special circumstances, without any such predisposition existing; as by confinement in ill-ventilated apartments, where numbers of persons are crowded together; bad food; want of exercise; uncleanliness, &c.; and we find that, under the same circumstances, diseases analogous to scrofula are generated in the inferior animals. It is certain, also, that by avoiding the exciting causes, an individual strongly predisposed to scrofulosis may avoid its development until late in life, or even altogether. We are compelled to admit, therefore, that although, in the vast majority of instances, there is an hereditary tendency to this disease, it may be produced without it, and that even when such tendency strongly exists, it may be prevented from developing itself by proper management.

The professor is disposed to believe, that the frequency of scrofula, in many eleemosynary institutions for children, is greatly owing to faulty architecture, by which proper ventilation is rendered impracticable. In such institutions, as in the Children's Asylum of the Philadelphia Hospital, the windows are not cut down to the floor so as to permit the fresh air to circulate freely, and the consequence is, that foul or impure air collects beneath the windows. In buildings intended for adults this would be of little moment, as their heads are constantly above or on a level with the window sill; but not so with children, who are, as it were, constantly immersed in the foul air that collects beneath the sill. It is probable, that if such faulty construction of the rooms were avoided, by extending the windows to the floor, many cases of scrofula might be prevented.

As before observed, it does not follow, that a person is scrofulous because he has inflammation or enlargement of the cervical ganglia; this is only an expression, at most, of the disease affecting the whole constitution. Scrofula has other expressions besides that affecting the glands, and among these a variety of ophthalmia is extremely common. 'Strumous ophthalmia,' of which numerous instances exist in the children's asylum in this Institution, presents several varieties. In all chronic cases of ophthalmia it is important to determine whether its chronicity may not be due to this diathesis, as it must influence the mode of treatment.

In one form, the edges of the eyelids are especially implicated, and become red, and covered with a yellow crust, the secretion of the inflamed Meibomian follicles. This is the ophthalmia tarsi. But perhaps a more common form is that in which phlyctænæ form on the conjunctiva, and, after continuing for a longer or shorter time, burst, leaving white cicatrices. The formation of the cicatrices often gives occasion to much uneasiness, from an apprehension that specks may be left, which may interfere with vision; but this dread is generally groundless. The professor has seen many cases of this character, some in his own family; but in no case has loss of sight resulted,—the white marks gradually disappearing. This form of ophthalmia is not unfrequently the consequence of some of the eruptive diseases, hooping cough, &c.

Otorrhœa is another affection extremely common in scrofulous persons, which consists in the discharge of a puriform matter from the ear. Sometimes the matter discharged proceeds only from the external ear. Not unfrequently, however, it flows from the middle ear, producing destruction of the ossicles, and perforating the membrana tympani. Although there may be this extensive destruction of parts, audition is not necessarily destroyed; yet it is usually greatly impaired. Loss of hearing may not, however, result, unless the membrane of the foramen ovale be destroyed also.

The professor himself has had an intermittent discharge from the middle ear from infancy, with, of course, perforation of the membrana tympani; but it has not destroyed, although it has impaired the hearing of that ear.

He has found no permanent benefit, in strumous otorrhœa, from local remedies. In every case the treatment—with the exception of repeated ablutions made by means of a syringe or otherwise—must be directed to the diathesis. Place the patient in a situation where fresh air may be enjoyed; send him to the sea shore when practicable; and put him upon the free use of animal food, and other means adapted to improve the condition of the blood. In these cases it will be found, as the general health and the plasticity of the blood increase, the otorrhœa will be diminished, and it may finally cease,—although such cases are generally very obstinate.

Sometimes scrofula manifests itself in disease of the osseous system, as in the morbus coxarius, white swelling, &c. [The professor here introduced a boy of markedly strumous diathesis, affected with coxalgia.] Since, then, scrofulosis has so many different expressions, how forcible is the objection to the definition before given, that it is essentially an enlargement of the cervical glands! Where the disease thus attacks the bony system, attention must, of course, be directed to the treatment of the local affection, but at the same time, it is all important to improve the constitution of the patient. The same may be said of glandular inflammations of a strumous character. The lecturer considers, that the enlargement of the glands is due to nutritive irritation, which has a similar effect as the irritation excited by the introduction of a thorn or other foreign body in any part of the tissues, and the effect of such irritation cannot be expected to cease until the cause is removed.

The professor remarked, that he dwelt long on this important subject, because it was generally little attended to, and imperfectly comprehended by students. In cases where many parts are simultaneously diseased, there is often much difficulty in determining, whether their obstinacy may not be dependent on this diathesis, and hence the importance of being acquainted with its characteristics, and, as far as practicable, with its nature.

The treatment of scrofulosis is both therapeutical and hygienical; but in most cases the greatest reliance is to be reposed on the latter. A judicious combination of both is, however, most satisfactory. All the remedies employed should be directed to the removal of the diathesis—to the improvement of the condition of the blood, so that it may produce euplastic materials rather than cacoplastic—and to a modification of the morbid state of the tissues.

General bloodletting is rarely had recourse to, as the vital actions are seldom exalted. Local depletion, acting as a revellent, is more employed. It is in scrofulosis, that the various articles comprising the class of eutrophics are administered, especially the preparations of iodine. Eutrophic, as the class are aware, is a term introduced by the professor to denote "agents whose action is exerted on the system of nutrition, without necessarily occasioning manifest increase in any of the secretions." They are articles that enter the mass of blood, and, by modifying its condition, impress corresponding changes on the system of nutrition. Of these, iodine is the chief; and a combination of it with iron—as in the ferri iodidum—would seem peculiarly appropriate, as the indication is to give tone to the system, and modify the condition of the blood, and of the nutritive system;—the iron fulfilling the former, the iodine the latter indication. It should be remembered, however, that for much good to result, its use must be persevered in for a length of time. A great error with the young practitioner is—his becoming too often discouraged; giving up a remedy too soon, and resorting to something else. So long as the indications remain the same, if the remedy be capable of fulfilling them, it should be continued until it is clearly seen that no good is likely to result from its further use. The use of the ferri iodidum, or any other article, in scrofula, should be continued for at least one month.

Recently, a most nauseous article has been brought forward with the highest pretensions—the oleum jecoris aselli—which is found to contain a small portion of iodine, and which may prove beneficial by the oil furnishing a modified chyle, and by its iodinic constituent. But it is exceedingly difficult to obtain it; and as difficult, after it is obtained, to persuade the patient to take it. The professor thinks, that olive oil, combined with a small quantity of iodine, would be as beneficial as the cod liver oil. It would, moreover, possess the advantage of being more accessible and far less unpleasant. He proposes to adopt it in the management of the cases of scrofula in this hospital, and to report the results.

Bromine has also been employed in scrofula. It acts in the same manner as iodine.

Although iodine and its preparations not unfrequently prove beneficial, the professor has often found them unsuccessful. He cannot, indeed, recommend them strongly, from the results of his own experience. The same remark applies to their use in tuberculosis.

The objection to the use of mercury is, that its ordinary action is presumed to be to diminish the amount, or the plasticity, of the fibrin of the blood. At least, it is given with this view in diphtheritic inflammation. Possessed of such properties, it must obviously be contra-indicated in scrofula, in which the plasticity of the blood, as has been seen, is diminished. If this be admitted, it must be manifestly improper to administer any remedy that would further diminish it. Mercury, then, being of doubtful propriety, if not positively injurious, there is no necessity for administering, especially as there are articles superior to it.

It is very probable, that the extract of walnut, and similar articles recommended in scrofulosis, exert nothing more than a tonic agency.

The professor here presented two men of colour, to exhibit scrofulous enlargements of the glands of the neck, one of whom had lately suffered from variola. Several of the glands of his neck are enlarged, and filled with cacoplastic and aplastic matter, which, if permitted to remain, will cause ulceration of the integuments, and leave unseemly cicatrices. The other patient exhibited one of these glands in an ulcerated condition.

The local management of these suppurating glands properly belongs to the domain of surgery; but the professor thought proper to allude to one or two points connected with their treatment. When they manifestly contain purulent matter, they should be early and freely opened in the direction of one of the natural folds of the part to avoid a cicatrix. The fluid must then be pressed out. The class were cautioned against the protracted use of poultices in these and similar cases, which are apt to induce great loss of parts.

The treatment then of scrofulosis, it will be seen, consists essentially in the administration of remedies that are adapted for modifying the condition of the blood, and in the adoption of means that will improve the general health—as eutrophics, exercise in the open air, well ventilated apartments, nutritious diet, &c.

The local treatment, as already stated, belongs to the surgeon.

Scrofulosis is not, as some definitions would import, confined to individuals with light hair or fair skin; for it is not unfrequent in those with swarthy complexion and black hair, and is very common amongst the negroes of the south in its most aggravated forms.

The lecturer observed that he was always unwilling to permit the class to separate for the season, without directing their attention to this diseased condition.

Professor Dunglison next drew the attention of the class to a case of

DEATH FROM DROWNING, which had occurred during the week, in order to elucidate the general pathology of asphyxia, on which he had dwelt elsewhere. The gentlemen attached to the class of Jefferson Medical College would recollect his remark on the occasion alluded to,—that in consequence of the conversion from venous into arterial blood being arrested in the lungs, the radicles of the pulmonary veins receive black blood into them, which is not adapted for their sensibilities; a stagnation of fluid takes place in those vessels, and, as a consequence, black blood is found filling the pulmonary artery, the right side of the heart, the large veins, and the veins of organs in the vicinity of the heart more especially. These phenomena were well marked in a recent case, to the particulars of which he wished to attract their attention.

A woman, in full health, was observed intoxicated on the banks of the Schuylkill, about one hour before the body was discovered in very shallow water. She had not, therefore, remained long under water; but owing to her helplessness, she had never, probably, risen to the surface, so that the asphyxia was complete, and it offered a good example of the pathological appearances in such cases. The body was examined by Dr. Farquharson, one of the resident physicians—to whom he was indebted for the history of the case and morbid specimens—about sixteen hours after death.

The face was swelled, and of a mottled purple. The arms and thighs presented patches of discoloration; and a small quantity of whitish froth issued from the mouth, the amount of which was not increased by pressure upon the chest, although a small quantity of watery fluid escaped when the body was turned over.

On opening the chest, numerous old pleuritic adhesions were found, on the removal of which, and the consequent compression of the lungs, a discharge of watery froth took place from the mouth. All the parts of the pulmonary tissue were engorged with blood, and were much heavier, and of a darker red colour than in the normal state. The posterior portions of both lungs were more engorged hypostatically, or from position. The trachea and bronchial tubes contained the same kind of watery froth, or frothy mucus, as that which issued from the mouth. The liver was large, engorged, and of a bright red colour. The *right cavities of the heart and the coronary veins were filled with dark fluid blood. The left cavities were empty.* Such are the main phenomena occasioned by the mode of death—*asphyxia from drowning.*

The lecturer exhibited the genito-urinary organs of the female, which were, in some respects, abnormous.

The bladder was contracted to a small size, and evidently incapable of containing much urine; and its inner coat was red and rugous. It was not known that she had complained of any affection of the urinary organs, although the condition of the bladder sufficiently indicated that it could only have held a very small quantity of fluid.

The upper portion of the vagina was of a dark red or lees of wine colour; and if the female had been pregnant, the appearance would have been regarded as highly confirmative of the views of Jacquemin, Kluge and others, who regard it as a strong single evidence of pregnancy. The uterus was rather large. The mouth of the organ contained a thick reddish fluid. The cavity of the body and the cornua were of an intensely dark red colour, and contained a fluid of a darker colour than that in the os tincæ, and the tissue of the uterus was much engorged with blood. It was not improbable, that she was menstruating at the time of her death.

Both Fallopian tubes were engorged with blood; and one of them presented a tortuous or convoluted appearance, as if it contained small elliptical bodies. Two transparent cysts, one of the size of a large pea, the other about one-quarter of that size, hung down into the peritoneal cavity. They were attached to the same pedicle, which was about one inch long. The ovary on this side was of a white colour, hard, and closely attached to the uterus by a short ovarian ligament, and a stringy bond of union. The other ovary had upon its surface three or four fluctuating prominences, which, on being opened, proved to be small sacs containing a reddish limpid fluid. The remaining portion of the ovary was of a uniform dark red colour. They resembled in their character the transparent cysts above mentioned; and the lecturer remarked, that he every year met with them, where there had been no signs to indicate any morbid affection of the uterus, tubes or ovaries. Yet it could readily be understood, that these cysts might undergo, in certain situations, and under favorable circumstances, farther development, so as to give occasion to ovarian dropsy. At times they might have their origin in the infecund ova, which would seem to be thrown off periodically during menstruation.

The professor lastly exhibited to the class some morbid specimens with which he had been favoured by Dr. Keating, one of the resident physicians, who had also furnished him with the history of the case that afforded them.

A man, aged 61, had been a soldier, and received a bayonet wound in the trachea, between the thyroid and cricoid cartilages, the cicatrix of which was very apparent.

He had consulted Dr. Keating two or three weeks previous to his last illness respecting a soreness in his throat, which interfered very materially with deglutition, and prevented his taking solid food. He attributed this affection to the effects of the bayonet wound; but as 30 years had elapsed, and he had never suffered from it before, it was thought that there must be some other exciting cause.

Counterirritants were ordered to his neck externally, and a strong alum gargle. His bowels, which were costive, were kept open by means of mild laxatives, and he lived entirely on eggs. He experienced some relief from this treatment, and appeared to be recovering, until Tuesday, Dec. 17, when he was found suffering from great dyspnœa, and complaining of something in his throat which interfered with respiration. He was continually endeavouring to clear his throat, and his voice was that of a hoarse whisper, resembling, at times, that of a ventriloquist. His mouth and throat seemed clogged with a thick ropy mucus, which he was obliged to remove with his fingers, not having the strength to expectorate it. Forty grains of ipecacuanha were given in two doses, to enable him to discharge this mucus, which had the desired effect: he vomited a great quantity of it, and sank into a quiet sleep. He was also ordered brandy beaten up with eggs, as his pulse indicated great prostration. He passed a very comfortable night, and continued better till 4 o'clock, P. M., of the next day, when Dr. Keating was sent for, who found his breathing very much oppressed, pulse small and quick, and throat so clogged up with thick mucus, that he could scarcely distinguish what he said: his voice was that of a hissing whisper, and the mucus rattled very audibly in his throat. He now complained of a very severe pain and oppression under the sternum. Under the feeling that the first indication was to get rid of this inspissated mucus, every effort was used to induce vomiting, but in vain; the patient's powers gradually failed. He died about 2 P. M. on the following morning.

On examination, after death, the lungs—which were exhibited to the class—presented all the appearances of the third stage of pneumonia, being filled with pus. The upper part of the larynx was extensively ulcerated, and there was a large quantity of ossific matter deposited in the seat of the old bayonet wound. All the cartilages were ossified, and the epiglottis was enlarged and indurated: there was also slight ulceration in the upper part of the pharynx. The trachea was healthy, but filled with this thick mucus. All the other organs of the body were natural.

